# Internet-Based Cognitive-Behavioral Therapy for College Students With Anxiety, Depression, Social Anxiety, or Insomnia: Four Single-Group Longitudinal Studies of Archival Commercial Data and Replication of Employee User Study

**DOI:** 10.2196/17712

**Published:** 2020-07-23

**Authors:** Mark D Attridge, Russell C Morfitt, David J Roseborough, Edward R Jones

**Affiliations:** 1 Attridge Consulting, Inc Minneapolis, MN United States; 2 Learn to Live, Inc Minneapolis, MN United States; 3 School of Social Work University of St Thomas St Paul, MN United States

**Keywords:** anxiety, cognitive behavioral, college, depression, insomnia, mental health, social phobia, stress

## Abstract

**Background:**

The growing behavioral health needs of college students have resulted in counseling centers reporting difficulties in meeting student demand.

**Objective:**

This study aims to test the real-world voluntary use by college students of 4 digital, self-directed mental health modules based on a cognitive behavioral therapy clinical model. The findings were also compared with those of employee users.

**Methods:**

Archival operational data from Learn to Live were extracted for student users at 4 colleges and universities in the Midwest region of the United States (N=951). The inclusion criteria were having clinical symptoms at established levels of moderate or higher severity and the use of 2 or more of the 8 lessons of a program within a 6-month period. Unique users in each program included 347 for depression; 325 for stress, anxiety, and worry; 203 for social anxiety; and 76 for insomnia. Paired *t* tests (two-tailed) compared the average level of change over time on a standardized measure of clinical symptoms appropriate to each program. Cohen *d* statistical effect sizes were calculated for each program. Potential moderator factors (age, gender, preliminary comprehensive assessment, number of lessons, duration, live coach support, and live teammate support) were tested together in repeated measures analysis of variance models with covariates in the full sample. Follow-up survey data (n=136) were also collected to explore user satisfaction and outcomes. Select data from another study of the same 4 programs by employee users meeting the same criteria (N=707) were examined for comparison.

**Results:**

The percentage of users who improved to a clinical status of no longer being at risk after program use was as follows: stress, anxiety, and worry program (149/325, 45.8%); insomnia program (33/76, 43.4%), depression program (124/347, 35.7%); and social anxiety program (45/203, 22.2%). Significant improvements (all *P*<.001) over time were found in the mean scores for the clinical measures for each program: stress, anxiety, and worry (*t*_324_=16.21; *d*=1.25); insomnia (*t*_75_=6.85; *d*=1.10); depression (*t*_346_=12.71; *d*=0.91); and social anxiety (*t*_202_=8.33; *d*=0.80). Tests of the moderating factors across programs indicated that greater improvement was strongly associated with the use of more lessons and it also differed by program, by gender (males demonstrated more improvement than females), and by the use of live support (particularly coaching). Analyses of survey data found high satisfaction, improved academic outcomes, and successful integration into the university counseling ecosystem. The operational profile and outcomes of the college students were also similar to those of employee users of the same programs from our other study of employee users. Thus, this study provides a replication.

**Conclusions:**

Self-directed internet-based cognitive behavioral therapy mental health modules are promising as a supplement to traditional in-person counseling services provided by college counseling centers.

## Introduction

### Background

Adjusting to college life is a significant challenge for many students [1], as they could struggle with exposure to new forms of stress and new social demands. Students navigating these challenges may experience loneliness, may lack a sense of campus belonging, and can be vulnerable to experiences such as the first depressive episode [2]. The onset of many mental health conditions coincides with adolescence and young adulthood [3].

A 2019 national survey of 43,140 college undergraduate and graduate students in the United States found that 42% of females and 34% of males met the criteria for moderate or severe psychological distress [4]. The same study also documented the past year prevalence rates for diagnosed mental health conditions and determined that 28% of females and 13% of males had depression and that 22% of females and 15% of males had an anxiety disorder (including social anxiety). Graduate students may face additional stressors and risks for mental health issues [5]. For example, in the same recent national survey in 2019, 33% of graduate students [6] received psychological health services in the past year compared with 25% of undergraduate students [7].

Although not a mental health disorder, sleep issues also pose risks for young adults attending colleges and universities [8]. For example, 50% of college students in the United States reported getting less than the recommended 7 hours of sleep during nights of the school week [4]. Although sleep is important, insufficient sleep is of particular risk for students with bipolar disorder who have an acute sensitivity to circadian and sleep-wake cycle disruption (what Frank [9] calls “sleep disrupters”). This realization has led sleep researchers Walker and van der Helm [10] to refer to good quality sleep as “overnight therapy.” Some colleges now offer resources specifically to address student sleep problems, such as the College Sleep Center at the University of St. Thomas [11].

Experiencing mental health problems can also adversely impact success at school. A recent national survey [4] found that college students reported the following common behavioral health issues had negatively affected their academic performance in the past year: stress (38%), anxiety (28%), sleep difficulties (23%), and depression (22%). Other studies have also shown that depression among college students is linked to poor academic performance [12] and to dropping out of school [13].

### Technology-Based Mental Health Resources

The growing behavioral health needs of college and university students have resulted in college counseling centers reporting difficulties in meeting the increased student demand [14,15]. This context provided an opportunity to test if internet-based cognitive behavioral therapy (iCBT) tools might assist students in self-treating some of the most commonly occurring mental health conditions [16]. A related question was whether self-directed technology support services can supplement in-person clinical relationships and encourage the appropriate use of on-campus counseling services.

New types of technology-based resources feature self-directed digital tools that are accessible from a website or mobile device. Most of these tools are asynchronous and do not involve live interaction with a mental health professional, although some are supplemented with live coaching. Many of these computerized tools are based on principles and clinical strategies derived from cognitive behavioral therapy (CBT) [17]. The general advantages of such tools include providing users with greater access to therapeutic support, flexibility in accessing support anytime from anywhere with internet access, and a significantly lower cost than in-person services. The privacy of technology tools can also help offset social stigma and related barriers to help-seeking that confront young adults [18]. According to recent reviews of the literature, at least 89 studies have explored the use of technology-based tools for college students interested in seeking support for a range of mental health, stress, and behavioral concerns [19-21].

These reviews offer support for iCBT tools in general for being able to improve clinical outcomes, but they also call for further research to be done in real-world settings that go beyond the use of college students as convenience samples in academic research pilot studies for testing the general efficacy of tools. Few studies have used an applied study design with archival data to examine the naturally occurring experiences of voluntary users of commercially available iCBT tools for mental health issues. This emphasis on experimental over applied real-life contexts for research in this area is reflected in the literature in general and especially among investigations using college students as participants.

### Interventions: The Learn to Live Suite of Self-Directed Web-Based Tools

This applied archival study was conducted with operational data from voluntary, registered users of Learn to Live, a suite of digital CBT-based self-directed programs for behavioral health issues, including anxiety, depression, social anxiety, and insomnia. The programs were hosted on a single dedicated website [22] and were accessible from any internet-capable device. Participants entered a code specific to their college or university on the website to get access to the service. In addition to a wide range of educational content on the website, the user had the option to begin their online experience by taking a brief assessment covering 5 domains: anxiety, stress, depression, social anxiety, and insomnia. Standardized and validated clinical assessment tools were used for each domain. After reviewing the assessment results, participants then had the opportunity to enroll in 1 of 4 CBT-based programs. Alternatively, users could directly start a program without first taking a comprehensive assessment. The very small percentage of users who were identified as at-risk, based on self-harm questions on the preliminary assessment, were referred to a crisis center for immediate support.

Each program consisted of 8 lessons that contained a brief assessment of clinical severity (repeated every lesson; using the same self-report assessment tool from the comprehensive assessment), videos, animations, and web-based application of CBT tools. Each set of lessons was designed to be completed in order from 1 to 8, and a prior lesson had to be completed before the user could progress to the next lesson in the program. Homework and practice with the tools was optional but recommended between lessons. Key elements of each lesson for each of the 4 programs are listed in Table 1.

In addition, users could opt to receive individualized coaching, and if selected, could choose the preferred channel of communication, either email, text, or telephone, with the coach. The coaches were employed by the service provider, and every coach had at least a master’s level education in psychology or counseling.

Users could also select a person from their personal life to communicate with during the program use, serving in a supportive role called a *teammate*. These live supports are offered with the goal of adding a relational component beyond self-help as a form of activating natural social support [23]. Coaching support from real people while using iCBT programs has also been demonstrated to improve outcomes among college users of technology-based tools for behavioral health issues [21,24,25].

Support for these 4 programs from Learn to Live was obtained in an earlier study [26] that involved a sample of 707 employee users who worked with multiple employers (also located in the Midwest region of the United States). The employee study used the same study design, the same archival data collection processes, and the same general time frame as this study of college student users. However, the employee study examined the experiences of users at both subclinical and clinical statuses at the start of program use for symptom severity level. Relevant data from only the clinical status group of the employee users were reanalyzed and presented in this study for comparative purposes with the college student users who were all starting out at the clinical status level of severity.

**Table 1:** Intervention elements and assessment for each lesson for the 4 Learn to Live iCBT programs.

Lesson		Intervention elements	Assessment
**Stress, anxiety, and worry**			
	1	PMR^a^ + better life goals + stress and anxiety tracker	GAD-7^b^
	2	STEPP^c^ model + mini thought inspection + ANTs^d^	GAD-7
	3	Full thought inspection + 12 assumptions + precautions	GAD-7
	4	Flaw-facing + worry-facing + perfectionism	GAD-7
	5	Active problem solving + time snapshot + reflection moment	GAD-7
	6	Present awareness + worry time	GAD-7
	7	Assertiveness + boundaries	GAD-7
	8	Lessons learned toolbox	GAD-7
**Depression**			
	1	Depression profile + my better life goals + activity log	PHQ-9^e^
	2	Testable hypothesis + identify 30-minute exercise + precautions	PHQ-9
	3	STEPP model + mini thought inspection + ANTs	PHQ-9
	4	Forgiveness scripts + thought inspection + 12 assumptions	PHQ-9
	5	Active problem solving + learned helplessness	PHQ-9
	6	Alternatives to dwelling + sleep enhancement form	PHQ-9
	7	Assertiveness + boundaries	PHQ-9
	8	Lessons learned toolbox	PHQ-9
**Social anxiety**			
	1	Social anxiety profile + social life goals	SPIN-17^f^
	2	STEPP model + identifying thoughts	SPIN-17
	3	Thought inspection + hot thought	SPIN-17
	4	Self-defense tactics checklist + find out for myself	SPIN-17
	5	Full thought inspection + ANTs	SPIN-17
	6	Fear facing trials list + fear facing log + fear facing menu	SPIN-17
	7	Fear facing debrief + avoidance	SPIN-17
	8	Lessons learned toolbox	SPIN-17
**Insomnia sleep**			
	1	Sleep tracker	MOS-Sleep-6^g^
	2	Alternatives to lying awake + sleep drive + sleep scheduling + recommended bedtime	MOS-Sleep-6
	3	Sleep helpers form + sleep barriers	MOS-Sleep-6
	4	PMR + guided imagery + worry notebook	MOS-Sleep-6
	5	STEPP model + mini thought inspection + ANTs	MOS-Sleep-6
	6	Thought inspection + deep sleep	MOS-Sleep-6
	7	Present awareness + worry time	MOS-Sleep-6
	8	Lessons learned toolbox	MOS-Sleep-6

^a^PMR: progressive muscle relaxation.

^b^GAD-7: Generalized Anxiety Disorder 7-item scale.

^c^STEPP: situation-thought-emotion-performance-precautions.

^d^ANTs: automatic negative thoughts.

^e^PHQ-9: Patient Health Questionnaire 9-item scale.

^f^SPIN-17: Social Phobia Inventory 17-item scale.

^g^MOS-Sleep-6: Medical Outcomes Study Sleep 6-item scale.

### Objectives of the Study

This study featured a longitudinal, repeated measures research design with archival data for college student users of iCBT programs offered by the same commercial provider that supported 4 different clinical issues. The goals of this study were to obtain empirical answers to the following research questions (RQs):

- RQ1: What is the profile among college students based on demographic factors and utilization factors for the 4 Learn to Live web-based iCBT programs?
- RQ2: Are each of the 4 programs effective in reducing the level of clinical symptoms after program use?
- RQ3: Is the extent of improvement in clinical symptoms after program use moderated by demographic factors of the user (age or gender) or by operational factors (ie, use of preliminary comprehensive assessment, number of lessons, time period of use, use of live support from a coach or from a teammate, or use of multiple programs)?
- RQ4: Are these moderator effects (if any) similar, when tested within each of the 4 programs?
- RQ5: From the survey data collected following the intervention, what can be learned about the different sources of promotion of the services, the impact of use on college-related outcomes and attitudes, different aspects of the user experience with counseling, and the level of overall user satisfaction with the service?
- RQ6: How does the user profile and clinical outcomes of the 4 programs for college student users in this study compare with that of employee users from a different study?

## Methods

### Archival Data

The data for the study were from postsecondary students using the *Learn to Live* service. Users were made aware of the service as a benefit open to all students through a variety of on-campus digital and interpersonal promotional practices. There was no direct cost to the participants in this study, as access to the website with the programs was sponsored by each of the schools. Students participated voluntarily and were not paid for using the web-based tools. The study period spanned 3 years (from October 2016 through October 2019). The insomnia program was added to the service suite in November 2017 and therefore was available only for the most recent 2 years of the full study period.

### Ethical Considerations

The privacy of users was protected by having all program and survey data deidentified before being shared with the independent consultant who conducted all of the analyses. As this was an applied study of archival anonymized data collected from routine use of the service, additional informed consent from individual participants beyond their initial consent agreement in terms of use was not required. Project approval from a college or a university internal review board was also not required. This context is similar to other applied studies of commercial web-based programs [26,27]. The use and analysis of archival operational data in this manner is consistent with the published ethical guidelines of the American Psychological Association [28]. 

### Inclusion Criteria and Participants

The following 5 criteria were established to select users appropriate to the study goals: (1) users had to be from a customer of *Learn To Live* in the higher education market segment (ie, a college or university), (2) users were required to be at a sufficient level of clinical severity (*clinical status*) at the start of program use, (3) users needed to engage in at least 2 (or more) of the 8 lessons of a program, and (4) users should not have completed all 8 lessons of a program in a single day nor should the time period of use from the start date (either the comprehensive assessment or the first lesson) to the date of the last lesson have exceeded 6 months. Application of these criteria yielded a sample of 951 unique users.

The final criterion was that if a student had used multiple iCBT programs during the 3-year period, then the experience data from only one program were included. Of the sample that met all 4 of the above inclusion criteria, most participants (806/951, 84.8%) used only 1 program, with the remaining 15.2% (145/951) having used multiple programs. Using more than 1 program was defined as using at least 1 lesson of 2 or more different programs during the study period. More specifically, 122 (12.8%) students had used 2 programs, 19 (1.9%) students had used 3 programs, and 4 (0.4%) students had used all 4 programs. The average number of lessons *per program* varied between these groups with a decreasing number of lessons per any one program as the total number of programs used increased: Users of 1 program had an average of 3.44 lessons per program; users of 2 programs used had an average of 2.25 lessons per program; users of 3 programs used had an average of 1.80 lessons per program; and users of all 4 of the programs had an average of only 1.68 lessons per program. Note that at least one of the programs used by an individual who had used 2 or more programs, had to have a minimum of 2 lessons used.

The choice of which program’s data to use for each student with multiprogram status was based on several criteria. Listed in order of importance, these criteria included: (1) clinical status on the symptom assessment at the start, (2) a higher number of lessons used (two minimum), (3) earlier start date in the study period, and (4) a longer time period for program use. For example, if a student had met the first set of criteria for use of 2 programs and had a score above the clinical score cutoff for the depression program but also a score that was below the clinical score cutoff for the insomnia program, then the data for the former was retained but not of the latter. To continue, if a student was above the cutoff scores for the severity symptom measures for both programs, then the program that had more lessons was retained. For example, if the depression program had 5 lessons and the insomnia program had 3 lessons, then the depression program was retained. Application of these criteria resulted in the following final mix of participants with data from only one program used: Of the 951 total unique users in the study, 347 (36.5%) users for the depression program, 325 (34.2%) users for the stress, anxiety, and worry program, 203 (21.3%) users for the social anxiety program, and 76 (8.0%) users for the insomnia program.

### Clinical Symptom Measures

Each of these measures of clinical symptom severity is a published, reliable, and validated scale from the scientific literature. Within each program, the symptom measure was repeated in every lesson. These 4 measures were aggregated in the comprehensive assessment (along with a fifth measure of perceived stress).

#### Anxiety

The generalized anxiety disorder 7-item scale was used to assess symptoms of anxiety [29]. This is one of the most widely used screening and outcome tools available for anxiety and has been shown, in past research, to have adequate levels of reliability and validity [30,31]. Sample items include the following: (1) *feeling nervous, anxious or on edge* and (2) *not being able to stop or control worrying*. The instructions state: “Over the last 2 weeks, how often have you been bothered by any of the following problems?” Items are rated on a 0 to 3 scale. Ratings on the items were summed and scores were categorized into levels of severity: low 0-4, mild 5-9, moderate 10-14, and severe 15-21. The clinical status for anxiety was defined as moderate or higher (score of 10+). The severity mix for general anxiety in the sample was 52.6% moderate (171/325) and 47.4% severe (154/325).

#### Depression

The patient health questionnaire 9-item scale was used to assess symptoms of depression [32]. This scale has been used in hundreds of research studies and has well-established validity and reliability [33]. The instructions state: “Over the last 2 weeks, how often have you been bothered by any of the following problems?” Sample items included the following: *Little interest or pleasure in doing things* and *feeling down, depressed, or hopeless*. Items are rated on a 0-3 scale. Scores on the 9 items were summed and then categorized into levels of severity: minimal 0-4, mild 5-9, moderate 10-14, moderately severe 15-19, and severe 20-27. The clinical status for depression was defined as moderate or higher (score of 10+). The severity mix for depression in the sample was 32.3% moderate (112/347), 44.7% moderately severe (155/347), and 23.0% severe (80/347).

#### Social Anxiety

The social phobia inventory (SPIN) was used to assess symptoms of social anxiety [34]. Past research has shown SPIN to have adequate levels of reliability and validity [35]. The instructions state: “Select the answer that best describes how much the following problems have bothered you during the past week.” Scores on the 17 items are rated on a 0-4 scale. Scores were summed and categorized into 5 levels of severity: minimal 0-18, mild 19-30, moderate 31-40, severe 41-50, and very severe 51-68. The clinical status for social anxiety was defined as moderate or higher (score of 31+). The severity mix for social anxiety in the study sample was 36.0% moderate (73/203), 42.4% severe (86/203), and 21.6% very severe (44/203).

#### Insomnia

To assess symptoms of sleep disturbance and insomnia, the sleep scale from the medical outcomes study (MOS) developed by the Rand Corporation [36]. The MOS sleep scale has proved to have adequate levels of reliability and validity [37]. The 6-item short version used item numbers 4, 5, 7, 8, 9, and 12 from the original full 12-item scale. The instructions for the measure state: “How often during the past week did you…?” The items included the following: (4) *get enough sleep to feel rested upon waking in the morning?* (5) *awaken short of breath or with a headache?* (7) *have trouble falling asleep?* (8) *awaken during your sleep time and have trouble falling asleep again?* (9) *have trouble staying awake during the day?* and (12) *get the amount of sleep you needed?* The recall period was slightly modified for use by Learn to Live, such that in the preliminary comprehensive assessment, the instructions for this scale used the *past 4 weeks* reference time period, whereas in each lesson of the program, the instructions had a reference time period of the *past week*. The 6 items were rated on a scale of 1-6. The ratings were weighted (1=0, 2=20, 3=40, 4=60, 5=80, and 6=100) and summed. The total score was then categorized into 4 levels of severity: minimal 0-29, mild 30-43, moderate 44-60, and severe 61-100. The clinical status of insomnia was defined as moderate or higher (score of 44+). The severity mix of insomnia in the study sample was 55.3% moderate (42/76) and 44.7% severe (34/76).

### Sources of First and Last Scores on Outcome Measures

The data source for the score at the start of program use was most often the student's score from the preliminary comprehensive assessment, which had been completed by almost 9 out of every 10 participants (829/951, 87.2%). For those who did not complete the comprehensive assessment, the score for the start of program use was taken from the symptom assessment done as part of the first lesson. The data source for the last score at the end of program use was taken from the student's score on the last lesson used, which varied within person from lesson 2 to lesson 8. Each program had a full range of lessons represented from 2 to 8.

### Follow-Up Survey

All registered users of the *Learn to Live* services were sent an email and invited to complete a self-report survey about their experiences. Modest financial incentives were provided to students who participated in a follow-up survey. Note that offering incentives for survey completion was a routine component of business operations and not a procedure unique to the research study. The specific questions and response options and the findings are presented later in this paper. A total of 136 users completed a survey during the valid follow-up period, defined as at least 1 month but not more than 6 months after the date of the last use of the program (between 31 and 183 days after the date of the last lesson used). About 1 out of every 8 students in the study (136/951, 14.3%) completed a valid follow-up survey.

Preliminary tests were performed by comparing the survey sample with the others in the total sample to determine the representativeness of the survey group. Specific statistical results for each of the measures compared are shown in Multimedia Appendix 1. The survey group was similar to the nonsurvey group on factors of age, completing the comprehensive assessment, and using a teammate. However, the survey group differed from the nonsurvey group in that it had more females, used more lessons, had a longer timer period of use, had more who used a coach, and had more who had tried more than one of the programs. Although those who participated in the follow-up survey process had greater engagement with the program, both groups had nearly identical clinical outcomes. Thus, the survey sample was considered representative of each program and the overall program use experience, despite some differences between the respondents and nonrespondents.

### Data Analysis

All analyses were conducted using SPSS version 25 [38]. Descriptive and inferential tests were performed as appropriate to the data and research questions. Details on the specific analyses performed are presented in the Results section.

## Results

### Part 1: Profile of College Student Users of iCBT Programs

This part of the paper describes the profile of college students based on demographic factors and their use of the 4 programs.

#### Demographic Profile of the Total Sample

The sample included 951 students from 4 colleges and universities, all located in the Midwest region of the United States. In the total sample, the average age of the users was 23.38 years (SD=6.54) and ranged from 15 to 62 years, although most users were in the 18 to 21 age group, typically of the college undergraduate experience. Females comprised the vast majority of program users at 74.2% (706/951), males were 23.6% (224/951), and 2.2% (21/951) of users self-identified as *gender diverse*.

Other background factors of race and year in college were only collected in the follow-up survey (described later in the paper, *n*=136). For race, 80.1% (109/136) identified as white, 8.1% (11/136) as Asian, 3.7% (5/136) as black or African American, 1.5% (2/136) as Hispanic or Latino, and another 6.6% (9/136) as *Other* or *no answer*. Most of the users who completed the survey were undergraduate students (85/136, 62.5%), although slightly more than a third were graduate students (51/136, 37.5%). Among the 85 undergraduates, the mix of school class was 15 freshman, 31 sophomores, 18 juniors, and 21 seniors. This profile indicates that these iCBT programs appealed to both undergraduate and graduate students who had a profile for age, gender, and race, which was consistent with the larger college student population in the United States [4].

#### Profile of Program Utilization in the Total Sample

This profile of program use is for the total sample (*N*=951) across the 4 programs. The average number of lessons used was 3.63 (SD=2.08) out of the 8 possible. The mean number of days of use from the start to the last lesson was 41.55 days (SD=43.77), with a median of 25 days and a range of 1 to 183 days. The period of time between the use of each lesson averaged 11.65 days (SD=13.14).

As expected, the number of lessons used and the duration of the period of use were strongly positively correlated (*r*=0.47; *P*<.001). The number of lessons used was not related to gender (*r*=−0.02; *P=.*54) but was somewhat correlated with age (*r*=0.14; *P*<.001), in that older students used more lessons. The duration of use was not related to gender (*r*=−0.01; *P=.*98) but was somewhat correlated with age (*r*=0.11; *P*<.001), in that older students had a longer period of use.

About 1 in every 5 students chose to involve a *coach* from the program staff for ongoing support during use of the program (209/951, 22.0%). The use of a coach was strongly associated with participating in a greater number of lessons (1.49 more lessons on average than when not using a coach; *r*=0.30; *P*<.001) and with greater duration of program use (37.13 more days of use on average than when not using a coach; *r*=0.35; *P*<.001). In contrast, the use of a coach was only weakly associated with demographic factors of older age (*r*=0.08; *P*=.02) and was unrelated to gender (*X^2^_1,951_*=0.01; *P*=.76).

About 1 in every 8 students chose to engage a *teammate* (a personal friend/family member) for ongoing support (124/951, 13.0%). The use of a teammate was not associated with the number of lessons used (*r*=0.05; *P*=.12), the duration of use (*r*=0.01; *P*=.72), or age of the user (*r*=−0.01; *P*=.81). However, the use of a teammate was strongly associated with gender (*X^2^_1,951_*=8.2; *P*<.001), such that females (102/706, 14.4%) were twice as likely to have used a teammate than were males (16/224, 7.1%).

#### Comparison of the Four Programs Based on User Demographic and Operational Factors

The demographic factors of age, gender, and operational use factors were also compared between the 4 iCBT programs. The results revealed significant differences between programs on 7 of the 9 factors tested (Table 2).

**Table 2:** Comparison of user demographic and operational factors: The Learn to Live program (N=951).

Factor			iCBT^a^ program				Test of differences				*P* value	
			Stress, anxiety, and worry	Depression	Social anxiety	Insomnia (sleep)	*F* test (*df*)		Chi-square (*df*)			
Number of users			325	347	203	76	N/A^b^		N/A		N/A	
**User demographic factors**												
	**User age (years)**							3.6 (3,947)		N/A		.01
		Median	21	21	20	22						
		Mean (SD)	23.24 (5.51)	23.21 (6.78)	23.00 (6.38)	25.72 (9.06)						
	**User gender, n (%)**							N/A		25.5 (6,951)		<.001
		Female	360 (80.0)	259 (74.6)	129 (63.5)	58 (76.4)						
		Male	63 (19.4)	76 (21.9)	70 (34.5)	15 (29.7)						
		Gender diverse	2 (0.6)	12 (3.5)	4 (2.0)	3 (3.9)						
**Operational factors of program use**												
	Comprehensive assessment: Yes, n (%)		286 (88.0)	309 (89.0)	165 (81.3)	69 (90.8)	N/A		8.5 (1,951)		.04	
	**Lessons used, n (%)**							2.0 (3,947)		N/A		.12
		2	152 (46.8)	152 (43.8)	83 (40.9)	39 (51.3)						
		3	52 (16.0)	69 (19.9)	52 (25.6)	9 (11.8)						
		4	41 (12.6)	27 (7.8)	31 (15.3)	7 (9.2)						
		5	24 (7.4)	20 (5.8)	10 (4.9)	3 (3.9)						
		6	11 (3.4)	13 (3.7)	11 (5.4)	2 (2.6)						
		7	7 (2.2)	8 (2.3)	5 (2.5)	1 (1.3)						
		8	38 (11.7)	58 (16.7)	11 (5.4)	15 (19.7)						
		Mean (SD)	3.58 (2.03)	3.80 (2.23)	3.37 (1.69)	3.78 (2.37)						
	**Number of days**							6.0 (3,947)				<.001
		Median	30	28	22	16						
		Mean (SD)	46.54 (46.82)	44.17 (44.77)	33.34 (38.30)	30.07 (33.83)						
		Range	1-182	1-183	1-163	1-143						
	Coach used: Yes, n (%)		84 (25.8)	79 (22.8)	33 (16.3)	13 (17.1)	N/A		7.9 (3,951)		.05	
	Teammate used: Yes, n (%)		42 (12.9)	77 (22.2)	2 (1.0)	3 (3.9)	N/A		57.2 (3,951)		<.001	
	Multi-user: Yes, n (%)		36 (11.1)	64 (18.4)	28 (13.8)	17 (22.4)	N/A		10.4 (3,951)		.02	
	Survey at follow-up: Yes, n (%)		37 (11.4)	54 (15.6)	34 (16.7)	11 (14.5)	N/A		3.7 (3,951)		.30	

^a^iCBT: internet-based cognitive behavioral therapy.

^b^N/A: not applicable.

##### Demographics Compared by Program

Both demographic characteristics of users differed significantly between programs. The average age of the students in the insomnia program was about 3 years older than the average age of the students in each of the other 3 programs. Although females were the majority of users in every program, the social anxiety program had relatively fewer women among users (64%) than the gender mix in the other 3 programs (range 75%-80%; female).

##### Operational Factors Compared by Program

The 4 programs were similar in the average number of lessons used per person. The percentage of users who completed a valid survey at follow-up after use was also similar across the 4 programs. However, significant differences between programs were present in 5 other utilization factors (Table 2). The percentage of users who had completed the comprehensive assessment was lower in the social anxiety program than in the other 3 programs (81% vs 88%-91%). The average period of use differed between programs, with the stress, anxiety, and worry and depression programs both having longer average periods of use (roughly 12 days more) than the social anxiety and insomnia programs. The stress, anxiety, and worry program and the depression program both had a higher coaching use rate than the social anxiety and insomnia programs (26%, 23% vs 16%, 17%, respectively). The depression and stress, anxiety, and worry programs both had a much higher use of teammates for support than did the insomnia and social anxiety programs (22%, 13% vs 4%, 1%, respectively). The percentage of students who used multiple programs during the 3-year study period differed significantly between programs (range 11%-22%).

### Part 2: Improvement in Clinical Symptoms by Program

This part of the results examines the primary outcomes of the study for change in the clinical symptoms among users in each program separately. Changes from before to after program use in the level of severity of clinical symptoms were empirically examined in two ways. The first approach was more clinically focused and determined how many cases changed from being in clinical status at the start (100% of users by design) to no longer being in clinical status after use. The second approach compared the average levels of symptom severity across all cases in each program before and after use. Both approaches were performed separately for each of the 4 programs. See Table 3 for details of the findings.

**Table 3:** Users at clinical status at start and last use and average level of clinical symptoms at start and at last use: by the Learn to Live program.

Characteristics			Internet-based cognitive behavioral therapy program								
			Stress, anxiety, and worry		Depression		Social anxiety		Insomnia (sleep)		All
Users, n			325		347		203		76		951
Measure			GAD-7^a^		PHQ-9^b^		SPIN-17^c^		MOS-Sleep-6^d^		N/A^e^
Score range			0-21		0-27		0-51		0-100		N/A
Clinical level score			10+		10+		31+		44+		N/A
Clinical status at start, %			100		100		100		100		N/A
**Test 1 of reduction in symptom severity at individual user level**											
	No change: Stayed clinical at last use, n (%)	176 (54.2)		223 (64.3)		158 (77.8)		46 (56.4)		N/A	
	Changed from clinical at start to subclinical at last use, n (%)	149 (45.8)		124 (35.7)		45 (22.2)		33 (43.4)		Unweighted average: 36.8%	
**Test 2 of reduction in symptom severity level average across all users**											
	Start, mean (SD)	14.50 (3.09)		16.44 (4.12)		43.89 (8.55)		60.46 (11.32)		N/A	
	Last use, mean (SD)	10.19 (5.02)		12.34 (6.41)		37.06 (13.31)		47.03 (18.43)		N/A	
	Improvement, %	29.7		24.9		15.6		22.2		Unweighted average: 23.1%	
Paired *t* test (*df*)^f^			16.21 (324)		12.71 (346)		8.33 (202)		6.85 (75)		N/A
Paired correlation, *r*			0.38		0.41		0.50		0.42		N/A
Effect size, Cohen *d*			1.25		0.91		0.80		1.10		N/A
Effect size level			Large		Large		Large		Large		N/A

^a^GAD-7: Generalized Anxiety Disorder 7-item scale.

^b^PHQ-9: Patient Health Questionnaire 9-item scale.

^c^SPIN-17: Social Phobia Inventory 17-item scale.

^d^MOS-Sleep-6: Medical Outcomes Study Sleep 6-item scale.

^e^N/A: not applicable.

^f^All *P* values <.001.

#### Change in Clinical Status by Program

For each program, the number of cases that were still above the established scale score cutoffs for *clinical* status at the last lesson used was examined. The results showed that the percentage of users who changed from clinical status at the start to become lower and subclinical in their level of severity after use was 46% for stress, anxiety, and worry, 43% for insomnia, 36% for depression, and 22% for social anxiety. Averaged across the 4 programs (unweighted by different sample sizes), 37% of the students changed from clinical to non–clinical status after use.

#### Change in Average Level of Symptom Severity by Program

Paired *t* test (two-tailed probability) and statistical effect size (Cohen *d*) analyses were performed for each of the 4 programs to test the extent of change in clinical symptom severity on average across all users from before to after use. The results indicated that each program had a significant improvement in severity. The average improvement (as a percentage of score reduction) for each program was as follows: 30% for stress, anxiety, and worry, 25% for depression, 22% for insomnia, and 16% for social anxiety. Each of these results had a statistical effect size that was considered as *large* (*d* range 0.80-1.25). Averaged across the 4 programs (unweighted by different sample sizes), there was a 23% reduction in the level of severity of clinical symptoms after use.

### Part 3: Multivariate Tests of Moderating Factors of the Extent of Improvement in Clinical Symptoms After Program Use in Total Sample

The findings in Part 1 revealed many differences between the 4 programs in terms of user characteristics and how the programs were used. Overall, there were also some correlations between many of the operational factors. This context indicated that the overall results found in Part 2 for clinical improvement specific to each program may be influenced by user demographics and operational factors. Given the different profiles of the 4 programs, it made sense then for us to explore changes over time in the clinical outcomes using other more sophisticated tests that take into account the joint influences of the user demographic and program use characteristics. This part of the results examined the impact of relevant potential moderator factors on the change in clinical outcomes in the full sample, considering all programs together.

As each of the programs had different outcome measures and a standardized measure was needed for analyses involving all programs in the same tests in the total sample, we used the difference scores for the change in symptom severity measures for each program (Multimedia Appendix 2). These difference scores when converted into percentages of change from first to last use ranged from 100% to −100% and had a near normal distribution of variance for each program such that some users had increased severity, some had little or no change, and some had decreased severity. The variation between the 4 programs in the extent of change in clinical outcome severity was statistically tested when controlling for all of the other user demographic and operational factors. The same tests also explored whether each of the demographic and operational factors were moderating the results for the extent of improvement after use, when controlling for the shared effects of the program used and all other factors. For example, was there a greater reduction in clinical symptoms for students who used more lessons in a program, all other factors being the same?

A repeated measures analysis of variance model with covariates (ANCOVA) was conducted with the dependent measure of the difference score for the amount of change in symptom severity from start to last use. The factors included the program (4 groups), the covariates of the demographic factors of user age (number of years) and gender (male, female, and gender diverse), the operational factors of use of the comprehensive assessment (yes/no), the number of lessons used (2-8), the time period of use (number of days), the use of a coach (yes/no), the use of a teammate (yes/no), and if the student had used more than one program (yes/no). Each continuous variable was used in the statistical tests as covariates with their full variance. The results for each factor are shown in the second column of Table 4.

However, subgroups of the continuous variables were also created for descriptive purposes to better understand the results using the estimated scores. This recoding process was done for the user, the number of lessons, the user’s age, and the duration of use. The number of lessons used was recoded into 3 categories: (1) lessons used 2, (2) lessons used 3-7, or (3) all 8 lessons completed. The age of the student was recoded into 2 categories: (1) those who were within the traditional undergraduate age range of 21 years or younger (*n*=534; mean=19.41 years, SD=1.11; median=19) and (2) those who were older and in the age range of 22 to 62 years (*n*=417; mean=28.46 years, SD=7.07; median=27).

The time period of use was recoded into 3 categories, based on dividing the sample into thirds (which was done separately within each program): (1) shortest time period, (2) middle time period, or (3) longest time period. As expected, these 3 groups differed in the total number of days of program use: (1) shortest time period (*n*=299; mean=5.20 days, SD=4.11; median=4 days), (2) middle time period (*n*=330; mean=26.13 days, SD=11.17; median=24 days), and (3) longest time period (*n*=322; mean=91.11 days, SD=39.83; median=79 days). Essentially, this yielded 3 groups that used the program for time periods that lasted approximately 1 week, 4 weeks, and 13 weeks.

Adjusted mean scores on the dependent measure of the percentage change in clinical symptoms were then calculated for each subgroup within a factor. This process yielded estimated scores for the clinical change outcome measure for subgroups of a particular factor, while at the same time controlling for the influence of all of the other factors. The detailed descriptive results for adjusted percentage change levels for each subgroup are presented in the third and fourth columns of Table 4.

**Table 4:** Main effect tests of each factor on the outcome of reduction in clinical symptoms when controlling for other factors: total sample (N=951).

Factor		Results with all factors in the model^a^		Improvement from start to last use in clinical symptoms by subgroups of factor (%)	Sample, n	Relative odds for pairings of subgroups with most difference
		*F* test (*df*)	*P* value			
**Overall**		11.37 (11,950)	<.001			
	All program average			23.9	951	N/A^b^
**Comprehensive assessment**		<1 (1,950)	.81			Yes 1.04 X greater than No
	Yes			22.9	829	
	No			22.0	122	
**iCBT^c^** **program used**		7.42 (3,950)	<.001			Stress, anxiety, and worry program 1.83 X greater than social anxiety program
	Stress, anxiety, and worry			29.3	325	
	Depression			24.1	347	
	Insomnia			21.2	76	
	Social anxiety			16.0	203	
**Age of user^d^** **(years)**		3.01 (1,950)	.08			Older 1.14 X greater than college age
	Older age of 22+ years			25.7	417	
	College age			22.5	534	
**Gender of user**		3.42 (2,950)	.03			Male 1.40 X greater than average of female and gender diverse
	Male			28.7	224	
	Female			21.0	706	
	Gender diverse			19.9	21	
**Number of lessons used^d^**		43.56 (1,950)	<.001			All 8 lessons 2.41 X greater than 2 lessons
	All 8 lessons			43.1	122	
	3-7 lessons			24.5	403	
	2 lessons			17.9	426	
**Duration (days of use)^d^**		<1 (1,950)	.66			Longer 1.13 X greater than shorter
	Longer: 13 weeks			24.6	322	
	Middle: 3 weeks			25.3	330	
	Shorter: 1 week			21.7	299	
**Coach used**		2.90 (1,950)	.09			Coach yes 1.20 X greater than no
	Yes			27.5	209	
	No			22.9	742	
**Teammate used (*SAW or DEP program users only*)**		2.11 (1,671)	.15			Teammate 1.20 X greater than no
	Yes			30.8	119	
	No			25.7	553	
**Live support combined use**		4.53 (2,950)	.01			Both 1.70 X greater than neither
	Both coach and teammate			37.7	33	
	One live support			26.4	267	
	Neither used			22.2	651	
**Multiple program user**		1.05 (1,950)	.31			Multiuser 0.86 X less than no
	Used 2+ programs			20.1	145	
	No			23.5	806	

^a^Mean percentages of change for subgroup estimated after statistical adjustment for all other factors listed. Test for teammate use was smaller as only 2 programs had enough participants with the use of a teammate to qualify (N=672).

^b^N/A: not applicable.

^c^iCBT: internet-based cognitive behavioral therapy.

^d^Tested in an analysis of variance model as a covariate using the full range of continuous data available. However, the subcategories shown have estimated mean scores for descriptive purposes only.

#### Outcomes Overall

Overall, when adjusted for the program and all other factors, the typical college student user experienced a 24% reduction in the severity of clinical symptoms. This overall level of reduction in clinical outcomes was moderated to a significant degree (*P*<.05) by 3 factors. These 3 factors included the program topic, the number of lessons used, and the gender of the user.

#### Outcome Differences by Program

The programs significantly differed from each other in terms of the change in outcome level, all other factors being equal. The stress, anxiety, & worry program had the highest level of improvement in clinical symptoms per average user (29%), followed by the depression program (24%), the insomnia program (21%), and the social anxiety program (16%) as the lowest. Thus, the program effectiveness differed between the 4 programs by a range of 16% to 29%.

A comparison of these adjusted results from the multivariate tests with the unadjusted results (Table 3) indicated very small differences between the 2 test methods (ie, change in the percentage of users at clinical status after use vs. change in the average level of symptom severity for all students after use). For the stress, anxiety, and worry program, 29.7% versus 29.3% equals a raw difference of 0.4 and a relative difference of 1.5%. For the depression program, 24.9% versus 24.1% equals a raw difference of 0.8 and a relative difference of 3.2%. For the insomnia program, 22.2% versus 21.2% equals a raw difference of 1.0 and a relative difference of 4.5%. For the social anxiety program, 15.6% versus 16.0% equals a raw difference of 0.4 and a relative difference of 2.6%. Thus, the overall results specific to each iCBT program did not change significantly, when taking into consideration the influences from the set of other relevant user and use context factors as well. This small difference between the testing methods also suggests that the other factors were acting in similar ways within each of the programs in how they affected the outcomes. However, this interpretation is examined directly in Part 4 of the results.

#### Outcome Differences Moderated by the Number of Lessons Used

The largest difference among the covariates was found for the operational factor of the number of lessons used. After controlling for which program was used and for all of the other operational and demographic factors, the students who completed the full program had an average improvement of 43%, compared with a 25% improvement among the students who used between 3 and 7 lessons, and only an 18% improvement for the students who used just 2 lessons. This was a relative difference such that the clinical success rate was 2.4 times higher for program completers than for those who participated minimally in doing only the first 2 lessons. This factor represents the level of dosage of the full content and intervention material. As this was the largest effect of all of the moderator factors tested, it was explored in more detail at each of the 7 specific levels of the number of lessons used (Multimedia Appendix 3).

**Figure 1:**
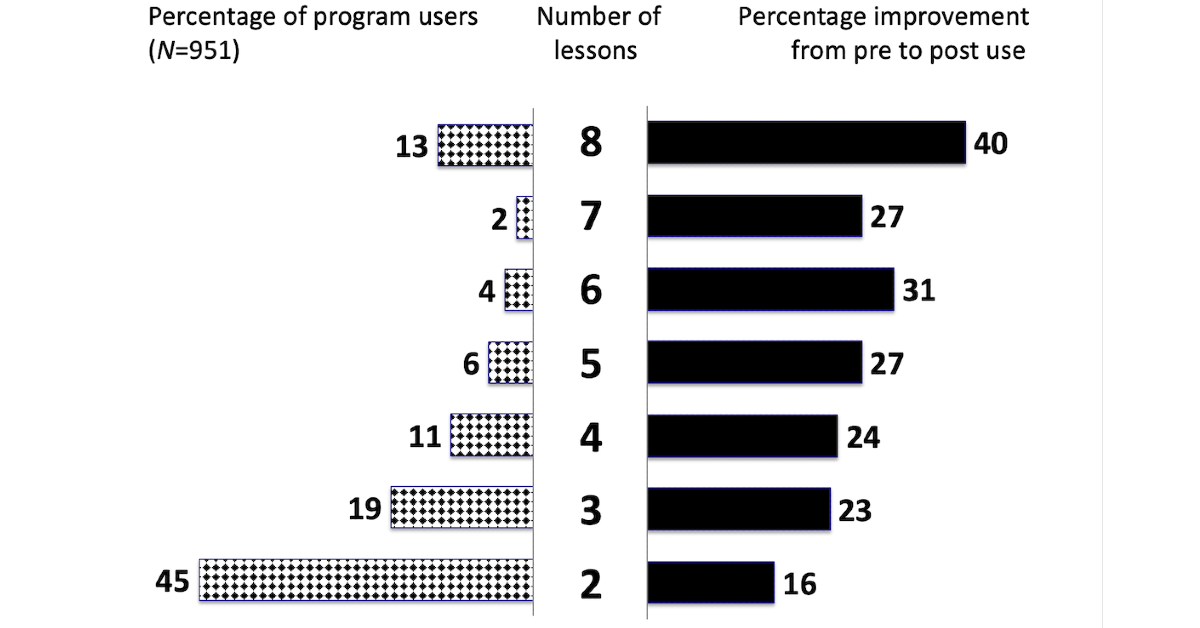
Results in total sample across internet-based cognitive behavioral therapy programs for moderating effect of greater number of lessons used (center) associated with greater improvement in clinical symptom severity (right side) and percentage of users at each level of lessons (left side).

The general pattern of how the number of lessons used was associated with clinical change and distribution in the total sample is presented in Figure 1. This graphic shows 2 bar graphs side by side and sharing a center column for the number of lessons used from a low of 2 to a high of 8. The bar chart on the left side shows how many students in the total sample used each total number of lessons (as a percentage). This figure reveals that most of the students (45%) had used 2 lessons, with the percentage of students in the middle levels of the number of lessons (from 3 to 7) being between 2% and 19% of all users, and only 13% of students who had completed all 8 lessons. The other bar chart on the right side shows the average percentage of improvement over time in clinical symptoms at each level of number of lessons used. The lowest level of improvement at 16% was for the students who used the fewest lessons (2), whereas the highest level of improvement at 40% was for the students who had used the most lessons (8), with the levels of improvement being in between these low and high ranges for the other students who had completed a number of lessons in between these extreme groups (3-7 lessons used). Thus, the impact on the extent of change in clinical symptoms was greater in an almost linear fashion as the number of lessons increased.

#### Outcomes Moderated by the Demographic Characteristics of the User

The gender of the user was significant as a covariate when controlling for all other factors (Table 4). Males had a higher level of improvement (29%) than did either females or gender diverse students (21% and 20%, respectively). When tested more specifically, with just 2 groups of males compared with the combination of females and gender diverse students as one group, controlling for all other factors, the result was even stronger for males, having a greater improvement by 1.4 (*F*_1,950_=12.95; *P*<.001). Older students had slightly more clinical improvement after use than did the traditional college-age students (26% vs 23%; *P*=.08).

#### Outcomes Moderated by the Use of Live Support

The use of live support only approached significance as a moderator when controlling for other factors (Table 4). Students who used a live coach from the service for support had slightly more clinical improvement after use than did the students who did not (28% vs 23%; *P*=.09). When tested combing the data from the only in the 2 programs with enough users of a teammate to provide a fair test (ie, the stress, anxiety, and worry and the depression programs), the students who had used a friend or family member for support had slightly more clinical improvement after use than the students who did not (31% vs 26%; *P*=.15).

However, when the 2 live support options were categorized into a combined variable of 3 groups (users of both coaching and teammates: 3.5% of all users; users of either kind of support: 28.0%; and nonusers of live supports: 68.5%), the findings were significant (*P*<.001) when controlling for all other covariate factors. These results indicated that the users of both live supports had the most improvement (38%), followed by the users of one of the live supports (26%), and with the non-users of live supports users having the least amount of improvement (22%).

#### Outcomes Moderated by Other Factors

Other operational factors did not significantly impact the extent of improvement in clinical symptoms once all other factors were considered (Table 4). These factors with no moderator effects included the duration of use, optional use of the comprehensive assessment before starting the first lesson, and whether or not the student had used multiple iCBT programs.

An explanation for why the duration of use was not significant is that it was positively correlated with the much stronger factor of the number of lessons used and thus it had minimal impact when the number of lessons factor was also included in the same analysis. The lack of effect for the comprehensive assessment is perhaps due to the timing of completing the comprehensive assessment, which is often done on the same day as starting the first lesson. Thus, the first lesson and the comprehensive assessment tend to have very similar scores on the clinical symptom measure used as the starting score.

Using more of the lessons on outcomes was critical for improvement. The lack of effect for the factor of multiple program user status makes sense when the average number of lessons *per program used* was lower by about 1 lesson among multi-program users than for single-program users (n=145, mean=2.14, SD=2.57 vs n=806, mean=3.44, SD=1.9, respectively).

In addition, both the comprehensive assessment and multi-program user factors had imbalanced distributions, with the vast majority of users being in 1 of the 2 categories (n=829 vs n= 122; n=806 vs n=145, respectively). Nonequivalence of sample sizes between groups can also affect the fidelity of the comparison tests.

### Part 4: Multivariate Tests Within Each Program of Moderating Factors of the Extent of Improvement in Clinical Symptoms After Program Use

Finally, additional repeated measures analysis of variance model tests with covariates were conducted to determine if the demographic and operational factors had moderator effects that were similar within each of the specific programs concerning their influence on the changes in the average level of symptom severity. The insomnia program, however, was excluded because it had too few users to allow us to conduct reliable tests of scores in the various subgroups of the factors. The analysis sample was also slightly smaller in each program tested, as the gender diverse students were excluded as they represented too small a group within each program for a reliable test (gender diverse: n=2 for stress, anxiety, and worry program; n=12 for depression program; n=4 for social anxiety program).

#### Stress, Anxiety, and Worry Program: Moderator Tests

For the stress, anxiety, & worry program (*N*=323), the results of the statistical analysis found that 2 of the 8 factors were significant as moderators of clinical symptom reduction after use (Multimedia Appendix 4). The effect for the lessons used was the largest of all the factors tested. Students who had used 2 lessons had the lowest level of clinical improvement at 23%, students in the middle levels of the number of lessons used (from 3 to 7) had a 31% improvement, and students who had completed all 8 lessons had the highest level of improvement with 46% reduction in symptoms. This was a relative difference such that the clinical success rate for the group of program completers was 1.9 times higher than the group that used just 2 lessons. The gender of the user was also significant when controlling for all other factors. Males in this program had a higher level of improvement than females (40% vs 27%, respectively). This was a relative difference such that the clinical success rate for males was 1.5 times higher than that for females. Other factors were not significant as moderators of improvement in the stress, anxiety, and worry program.

#### Depression Program: Moderator Tests

For the depression program (*N*=335), the results of the analysis found that 2 of the 8 factors were significant as moderators of clinical symptom reduction after use (Multimedia Appendix 4). The effect for the lessons used was the largest of all the factors tested. Students who had used 2 lessons had the lowest level of clinical improvement at 18%, students in the middle levels of the number of lessons used had a 24% improvement, and the students who had completed all 8 lessons had a 48% reduction in symptoms. This was a relative difference such that the clinical success rate for the group of program completers was 2.8 times higher than the group that used just 2 lessons. The use of a teammate among the depression program participants had a higher level of improvement than those who did not use a teammate (32% vs 23%, respectively). In addition, both demographic factors (age and gender) approached significance when controlling for all other factors among users of the depression program (*P*<.10). Males in the depression program had a slightly higher level of improvement than females (31% vs 23%, respectively). Older age students in the depression program had a slightly higher level of improvement than younger students (29% vs 22%, respectively). Other factors were not significant as moderators of improvement after use in the depression program.

#### Social Anxiety Program: Moderator Tests

For the social anxiety program (*N*=199), only one factor was significant in the statistical analysis as a moderator of clinical symptom reduction after use (Multimedia Appendix 4). The gender of the user was significant when controlling for all other factors. Males who addressed social anxiety had a higher level of improvement than females (21% vs 13%, respectively). The effect for the lessons used was in the same direction as in the other programs, yet failed to reach significance, and all of the other factors were not significant as moderators of improvement in the social anxiety program.

In summary, the findings for tests of moderator effects conducted separately within each program had a pattern of results that was mostly similar to the same tests conducted in the full sample across programs. The number of lessons used had the same pattern of findings in each program and was highly significant in 2 of the 3 programs specifically, although not for the social anxiety program (perhaps due to the smaller sample size). For gender, males improved more than females in each program. The effect of age approached significance in the depression and social anxiety programs but not in the stress, anxiety, and worry program. The effect of the use of teammate was found to be significant for the depression program but not for the stress, anxiety, and worry program. Coaching contributed to slightly better outcomes, but was not significant for any of the 3 programs tested. Other operational factors did not affect the outcomes within each program.

### Part 5: Survey Outcomes

For the survey data, a descriptive approach guided the data analysis to explore user responses concerning the promotion of the services, experience with counseling, impact on college–related outcomes and attitudes, and overall user satisfaction with the service. The items and responses are presented in Table 5. Students reported becoming aware of the services from a variety of sources within the college environment. The most effective promotional channel was campus email (45%), followed by on-campus printed signage (27%), campus health center (19%), on-campus digital signage (15%), the website of the college/university (13%), and a friend or classmate (4%).

Other questions on the survey asked about the impact of the service use concerning 9 different academic related outcomes. The first set of questions examined positive academic behaviors. The results found that users were more likely after use to ask for help needed for classes (40%), to participate in class (29%), to complete assignments (15%), to make presentations in class (15%), and to go to class on time (12%). When combined, about 8 in every 10 students had one or more of these positive academic outcomes. A second theme involved how various adverse academic outcomes were reduced or avoided after use of the service. Some students reported that they were less likely to drop out of school (20%), were less likely to transfer away (15%), were less at risk of dropping a course (14%), or were able to avoid academic probation (10%). Considered together, about 1 in every 3 students who used the service had avoided experiencing at least one of these adverse outcomes. Overall, almost 9 in every 10 student users were successful in one or more of the full set of 9 academic outcomes.

**Table 5:** Results of items on follow-up survey: data averaged across all programs (N=136).

Item			Users, n (%)
**Awareness of service:** **How did you learn about Learn to Live?**			
	*Campus email* ^a^		61 (44.9)
	*On-campus printed signage*		27 (27.2)
	*On-campus digital signage*		20 (14.7)
	*College website*		18 (13.2)
	*On-campus health clinic*		26 (19.1)
	*A friend or classmate*		6 (4.4)
**Academic outcomes:** **How has** **Learn to Live** **affected your college experience (check all that apply)?**			
	**Positive outcomes experienced**		
		*I am more likely to ask for help I need for classes*	55 (40.4)
		*I am more likely to participate in class*	39 (28.7)
		*I am more likely to complete assignments*	21 (15.4)
		*I am more likely to make presentations in class*	20 (14.7)
		*I am more likely to go to class on time*	16 (11.8)
		Sum: Any one of the above 5 positive outcomes (yes)	108 (79.4)
	**Adverse outcomes avoided**		
		*I am less at risk of dropping a course*	19 (14.0)
		*I have been able to avoid academic probation*	13 (9.6)
		*I am less likely to drop out of college/university*	27 (19.9)
		*I am less likely to transfer away from this college/university*	20 (14.7)
		Sum: Any one of the above 4 adverse outcomes avoided (yes)	50 (36.8)
		Sum: Any one of the above 9 outcomes (yes)	118 (86.7)
**Attitude toward college:** **Do you now have a more favorable attitude toward your college because they provide Learn to Live as a free benefit?**			
	*Yes*		103 (79.4)
	*No*		33 (20.6)
**Satisfaction:** **Overall, how satisfied were you with the Learn to Live experience?**			
	*Very satisfied*		28 (27.9)
	*Somewhat satisfied*		81 (59.6)
	*Somewhat dissatisfied*		14 (10.3)
	*Very dissatisfied*		1 (0.7)
	*Don't know*		2 (1.5)

^a^Italics indicate specific response options to the question.

The results of another item indicated that about 8 out of every 10 students had a more favorable attitude toward the school because the school provided the web-based counseling service. Improved academic–related outcomes from the use of the program may have contributed to a better overall opinion about the school. Finally, a key finding of the survey was that almost 9 out of every 10 users were satisfied with their experience with the web-based program.

The results of another item on the survey indicated that about half of the students (74/136, 54.5%), all of whom had met the clinical symptom threshold at the start of use, reported either a current or a past year experience with in-person counseling. Thus, about half of the students who used these digital mental health support tools already had experience with counseling from a live person. In addition, about a third of the users (52/136, 38.3%) were actively engaged in other in-person therapy at the time of their use of the iCBT program. This finding indicates that the use of the self-directed web-based support tool was adjunctive to in-person counseling for about 1 in every 3 of these students. In addition, among the subgroups of those who reported being currently involved with other therapies, half (26/52, 50.0%) reported that they were *getting more out of it* since adding the web-based resources. Thus, the use of the iCBT tools had a positive effect on their ongoing therapy experience.

The findings presented earlier on the importance of doing more of the lessons to achieve better outcome improvement were informed by responses on the survey for an item that asked why a student did *not* use all of the lessons in a program. This was answered by 74 of the 136 survey respondents. Not having enough time to participate more often was the most common response for the subgroup of users (37/74, 50.0%). About 1 in 6 of these noncompleters indicated that they had improved enough to feel that they could stop before using more lessons (12/74, 16.2%). Another 1 in every 6 of these students did not complete the program because they were not able to relate to the content or did not find the program was helpful (11/74, 14.9%). Taken together, these comments suggest that using fewer than the full 8 lessons was influenced more by a variety of nonsystematic factors unique to the students involved than it was to the program content or functionality.

### Part 6: Comparison of College Student and Employee Users—User Profiles and Outcomes

This part of the results examines the replication of this study with another similar study of employee users of the same iCBT resources. The primary findings of this study are similar to those found in a recent study of the same 4 programs when used by employees [26]. The sample that was starting program use above the clinical severity score thresholds was a total of 707 people. The sample sizes, demographics, and program use characteristics for the college and employee user samples are listed for each of the 4 programs in Multimedia Appendix 5. To simplify the comparison, the characteristics and results for the college users and the employee users were examined as simple averages across the 4 programs (ie, unweighted by the sample size differences in programs within each user sample, calculated as scores on a factor added up for the 4 programs and then divided by 4). These full study averages are presented in Multimedia Appendices 5 and 6.

For the demographic characteristics, the two samples were similar for gender (78% females in the employee sample) but, as expected, were quite different in age. The college sample users with an average age in the mid 20s were about 15 years younger than the average age of almost 40 years old for the employee user sample.

The two samples were quite similar in terms of the operational factors (see details in Multimedia Appendix 5). Both samples had most of the participants at the clinical level using the stress, anxiety, and worry or the depression programs more than the other two programs. However, the insomnia program had been used by a higher percentage of employee users than college users (18% vs 8%, respectively). The rate of completion of the comprehensive assessment before starting the program was the same at 87% for both of the samples. The average number of lessons used in the depression program was similar for the two samples, but the employee sample had a slightly higher number of lessons used in the other 3 programs compared with the student sample. Across all 4 programs, the average number of lessons used per program participant was similar, but slightly more for the employee users than the college users (yet this difference between the group averages was quite small - at less than one-half of 1 lesson). The average period of time used in each program was also similar for the two samples in each program. Comparison of the average across all 4 program for the period of use was also very similar, at only 1 day difference between the college and employee users (both at about 40 days).

The use of live support also had some modest group differences within each program (see details in Multimedia Appendix 5). The percentage of users with coach support in each program was greater for employees than it was for college students. The average across programs was 32% with coaches for employees compared to 21% with coaches for college students. The percentage of users with teammate support in each program was more similar for the two groups. The average across programs was 11% with teammates for employees and 10% with teammates for college students.

**Figure 2:**
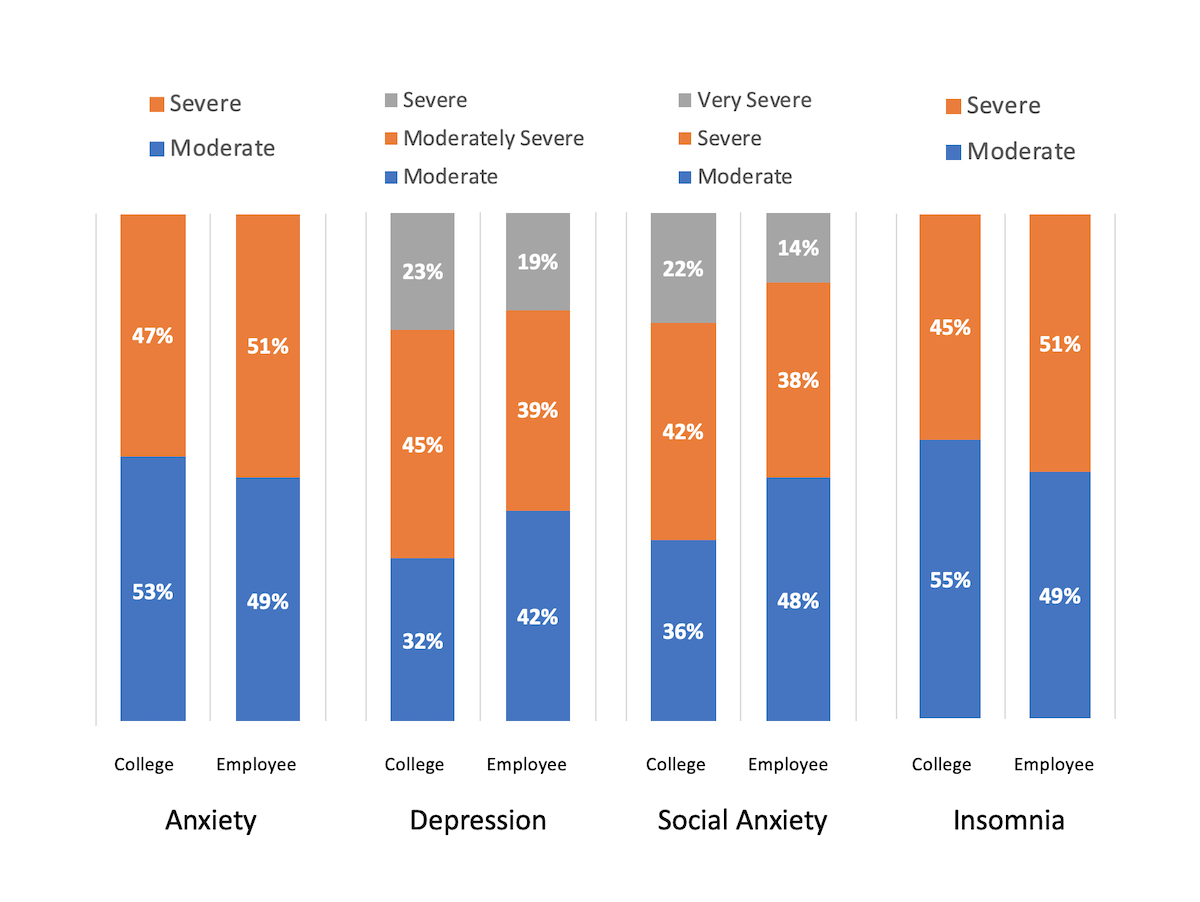
Comparison of percentage of college users and employee users of the same internet-based cognitive behavioral therapy programs at different levels of symptom severity at pre: by clinical assessment measure specific to each program.

By design, all users in both samples were above the cutoff scores for clinical status with moderate or higher levels of symptom severity. Depending on the measure, there were 2 or 3 levels of severity within the clinical status range. The data showed that the college and employee samples were also largely similar in the percentage of users at each severity level (Figure 2; with the statistical details in Multimedia Appendix 7).

**Figure 3:**
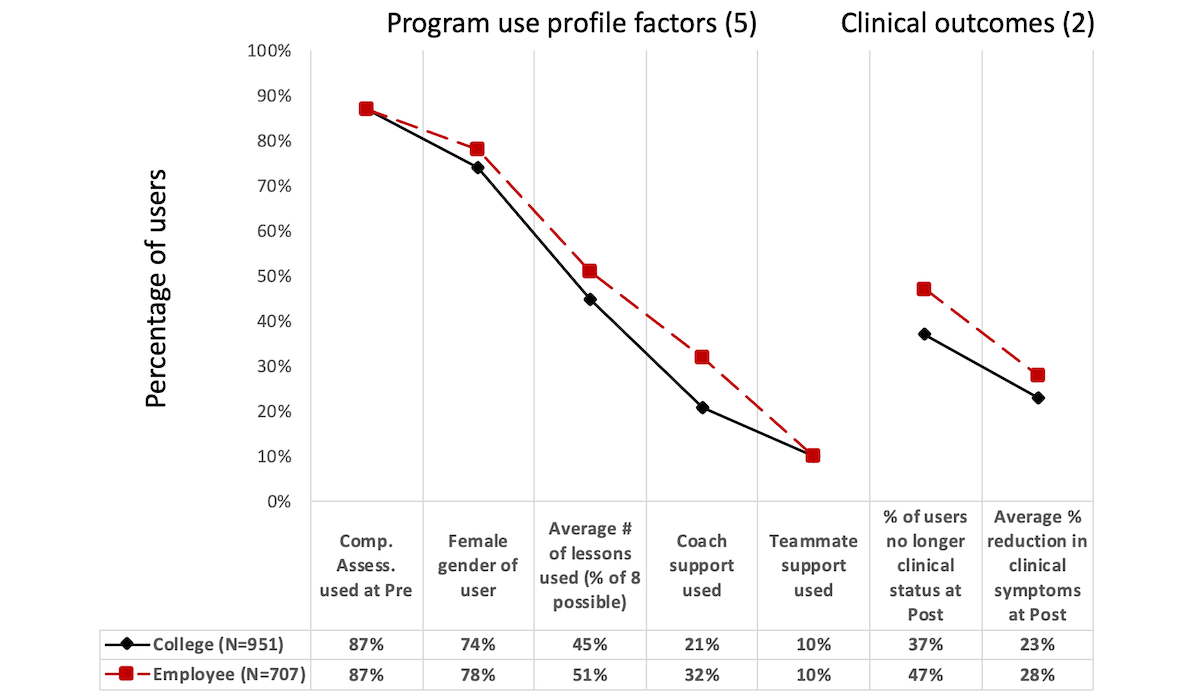
Comparison of college users and employee users of the same internet-based cognitive behavioral therapy programs on context factors and on clinical outcomes: both groups averaged across the 4 programs.

Considered together, the college student and employee groups were similar in most of the key factors. A summary of the user group averages is displayed in Figure 3. The source statistics for Figure 3 are provided in Multimedia Appendices 5 and 6. This high level of similarity offered a fair context to compare the clinical outcomes for each iCBT program for college and employee users.

The first outcome was how many of the users had changed from being at clinical status at the start of use to being below the threshold and no longer at clinical status after use. On average across the 4 programs (unweighted mean), 36.8% of the college users had this outcome compared to 46.8% of the employee users.

Next, the outcome of the average amount of change across all users in each sample was compared. The relative level of reduction in clinical symptoms for the typical user (as a percentage change from the mean score at the start to the mean score at the last use) was generally similar in each of the iCBT programs for the two samples. However, the employee users had a higher level of symptom improvement than the college users within each program. When averaged across the 4 programs (unweighted mean), the employee sample had an average of 28.9% reduction in symptom severity per person. This same metric was a 23.1% reduction for the college users. The results for clinical change by each program are presented in Multimedia Appendix 6.

Perhaps the levels of the moderating factors of the number of lessons used and the use of coaching, which were both somewhat higher for employee users compared with college users, contributed to the slightly better clinical results for the employee user sample. Both these factors were also significant moderators of clinical improvement when tested in the study of employees involving both clinical and subclinical users [26]. In summary, the comparison of the college user sample to the employee user sample revealed mostly similar profiles of user characteristics, operational use activity, and the primary clinical outcome results.

## Discussion

### Principal Findings

This study examined how different technology-assisted support tools can reduce symptoms associated with anxiety, depression, social anxiety, and insomnia. All 4 of these programs had significant reductions in clinical symptom severity after use, and each program had results of a large statistical effect. When averaged across the programs and also controlling for relevant demographic and operational factors, the typical user had a 24% reduction in symptom severity. At the individual level, about 1 in every 3 users changed from being in clinical status at the start of use to being at a nonclinical level of severity after use. Our findings replicate the generally positive results found in other studies of college student users of behavioral health digital tools on clinical symptoms [19-21,25].

Therapeutic *dosage* emerged as a particularly potent moderating variable, with much better results for those who participated fully in each program by completing all 8 lessons compared to those who participated minimally. Among program completers, the extent of improvement was *more than double* that of those who used only 2 lessons. This beneficial effect of adherence to the intervention (through completing more of the intended lessons) is a finding consistent with other research on adherence conducted in tests of similar kinds of iCBT tools for mental health issues [24,39-41].

Better clinical improvement among male users than among female users emerged as a surprise finding, even though less than a third of all users identified as male. One explanation could be the anonymity that this computerized medium affords and that there is no requirement to talk about one’s mental health challenges with another person. In this way, male users (especially those with social anxiety issues) may have felt less socially exposed by not having to talk with a counselor to better understand the nature of social anxiety. Male users may also be drawn more to the CBT oriented approach than to emotion-focused coping strategies, which are often emphasized in traditional talk therapy and which male college students tend not to prefer [42]. Gender differences in use rates and the effectiveness of technology-based support tools are also being explored in other research [43-45].

The use of optional live support was associated with an increased level of engagement in the key aspects of participation and yet was only weakly associated with better improvement in clinical symptoms, once these other more impactful factors were also considered in the same tests. In the total sample, however, the combination of using both live supports at the same time was significantly greater than using just one type of support or none at all. Coaching tended to be used more often and had a larger effect on the clinical outcome improvement than the options of live peer support from friends or family. The survey also asked participants to comment on their experience with using a coach. Two themes emerged from these qualitative comments as to why it was helpful. First, the coach provided accountability for engaging in more lessons and over a longer period of time. Second, the coach provided a caring, personalized form of support. The positive role of live coach supports found in this study has also been found in past research on technology tools for depression [46].

Optional peer supports of a friend or family member as teammates were used far less often than coaching support (and almost not at all in two programs). Note that coaches were also used more than teammates in the study of employee users of the same programs with the same pattern of very low use of teammates for the social anxiety and insomnia programs (see details in Multimedia Appendix 5). Teammate use was not associated with increased operational engagement. However, when a teammate was used, support from a personal contact had a small positive impact on the clinical change outcomes for the users in the depression program (but not in the other program with enough users of teammates to test). One potential explanation could be the preference among some college students to use social support from peers (ie, teammates in this study) when mental health issues are framed more as enhancing well-being and normal life challenges rather than as assisting in the treatment of clinical issues [47].

The survey data from the same users also revealed high levels of satisfaction and more favorable attitudes toward college or university. Program participation was also positively associated with at least one school performance outcome for 9 out of 10 students. About half of the students who used the web-based programs also had past year or concurrent experience in utilizing a face-to-face counselor. Moreover, one-fifth of the student users were referred to the digital services by the university counseling center, reflecting the acceptance of the digital services by the counseling centers and some existing integration of in-person and digital services. The benefits of using both traditional in-person and technological clinical supports have emerging research support [48].

The results of this study replicate the findings obtained from a recent study of employee users of the same set of web-based programs from Learn to Live [26]. The results of this study are similar to the findings in recent studies from other commercial providers of digital technologies designed to support common mental health and insomnia concerns among adults [25,27,39,49,50].

### Limitations

As in all applied research projects, there are certain limitations to this study. It was conducted using samples of college and university student users from multiple schools who had voluntarily used one particular commercial service. It is unknown whether these findings with this suite of tools can be replicated in other contexts of college student users. There were no comparison groups to assess the relative benefit from these iCBT tools compared to a matched group of nonusers. It is likely that some level of symptom reduction would occur naturally over time or form other causal forces not measured in this study. If so, whether the improvements over time in each program would have large size statistical effects if comparison groups of nonusers had been included in the study design is unknown. In addition, the causal mechanisms of how these web-based tools contribute to clinical improvements need further examination under more rigorous experimental study design conditions.

### Conclusions

This paper adds to the sophistication of the existing literature by comparing 4 distinct clinical topics, all of which shared the same digital platform and similar interactive website tools. Considered together, the variability in the number of lessons and in the total time period of use lends evidence to the flexibility that iCBT programs have to accommodate individual student preferences concerning when, how often, and how much to use the lessons. These iCBT tools appear to be associated with improved academic functioning and for some students, even the ability to stay in school. Additional study of outcomes beyond the typical focus on clinical symptoms in future studies would potentially add to the overall value proposition for web-based tools in support of the mental health of college students.

## Appendix

Multimedia Appendix 1 Comparison of survey and non-survey subsamples on demographic, utilization and clinical outcomes: Total sample across programs.

Multimedia Appendix 2 Psychometric profile of difference scores of change in severity of clinical symptoms from Pre to Post use: by Learn To Live program.

Multimedia Appendix 3 Source statistics for Figure 1. Percentage of program users and improvement in clinical outcome, both by number of lessons.

Multimedia Appendix 4 ANCOVA tests of moderator effect for demographic and operational factors on outcome of percentage reduction in clinical symptoms from pre to post (when also controlling for other factors): for three programs.

Multimedia Appendix 5 Comparison of college student users (this study) and employee users (other study) on age, gender, and operational use factors: by Learn To Live program.

Multimedia Appendix 6 Comparison of college student users (this study) and employee users (other study) on clinical symptom severity groups and change in severity from Pre to Post use: by Learn To Live program.

Multimedia Appendix 7 Source statistics for Figure 2 - Severity levels at start of use by program.

## Abbreviations

CBT: cognitive behavioral therapy
GAD-7: generalized anxiety disorder 7-item scale
iCBT: internet-based cognitive behavioral therapy
MOS: medical outcomes study
MOS-Sleep-6: medical outcomes study sleep 6-item scale
PHQ-9: patient health questionnaire 9-item scale
SPIN: social phobia inventory
SPIN-17: social phobia inventory 17-item scale
