# Supplementary material for: Internet-Based Cognitive-Behavioral Therapy for College Students With Anxiety, Depression, Social Anxiety, or Insomnia: Four Single-Group Longitudinal Studies of Archival Commercial Data and Replication of Employee User Study
Source: JMIR Form Res. 2020 Jul 23;4(7):e17712. doi: 10.2196/17712 (PMC7413280; doi:10.2196/17712)
Supplement: Multimedia Appendix 1 [file formative_v4i7e17712_app1.docx]

| Factor | Survey | Non-survey | Test of difference  & interpretation |
| --- | --- | --- | --- |
|  | *n*=136 | *n*=815 |  |
| **Gender of user % (*n*)** |  |  | *X*^2^_2_=7.1, *P*=.03  Survey more females |
| Female | 82.4 (112) | 72.9 (594) |  |
| Male | 14.7 (20) | 25.0 (204) |  |
| Gender Diverse | 2.9 (4) | 2.1 (17) |  |
| Age of user: years  – mean (SD) | 23.30 (6.46) | 23.39 (6.56) | *t*_950_<1, *P*=.88  Similar |
| Comprehensive assessment at start before use – Yes % (*n*) | 89.7 (122) | 86.7 (706) | *X*^2^_1_<1, *P*=.34  Similar |
| Number of lessons used  – mean (SD) | 4.22 (2.39) | 3.53 (2.01) | *t*_949_=-3.60, *P*<.001  Survey more lessons |
| Duration: days of use  – mean (SD) | 49.46 (40.54) | 40.23 (44.17) | *t*_949_=-2.28, *P*=.02  Survey longer time |
| Coach support used  – Yes % (*n*) | 33.1 (45) | 20.1 (164) | *X*^2^_1_=11.4, *P*<.001  Survey more coached |
| Teammate support used  – Yes % (*n*) | 14.7 (20) | 12.8 (104) | *X*^2^_1_<1, *P*=.53  Similar |
| Multiple programs used  – Yes % (*n*) | 21.3 (29) | 14.2 (116) | *X*^2^_1_=4.53, *P*=.03  Survey more multi-users |
| Percentage reduction from Pre to Post in severity level of clinical symptoms  – mean (SD) | 23.34 (0.29) | 23.85 (0.34) | *t_950_*<1, *P*=.87  Similar |
