# Supplementary material for: Internet-Based Cognitive-Behavioral Therapy for College Students With Anxiety, Depression, Social Anxiety, or Insomnia: Four Single-Group Longitudinal Studies of Archival Commercial Data and Replication of Employee User Study
Source: JMIR Form Res. 2020 Jul 23;4(7):e17712. doi: 10.2196/17712 (PMC7413280; doi:10.2196/17712)
Supplement: Multimedia Appendix 2 [file formative_v4i7e17712_app2.docx]

|  | iCBT^a^ Program | | | | All |
| --- | --- | --- | --- | --- | --- |
| Descriptive statistics for difference score of clinical symptom severity^b^ | Stress, anxiety, and worry | Depression | Social  anxiety | Insomnia  (sleep) |  |
| Number of users | 325 | 347 | 203 | 76 | 951 |
| Source measure | GAD-7^c^ | PHQ-9^d^ | SPIN-17^e^ | MOS-6^f^ |  |
| Median | .294 | .235 | .080 | .208 | .200 |
| Mean | .291 | .245 | .154 | .219 | .240 |
| SD | .329 | .366 | .262 | .208 | .331 |
| Skew | -.13 | -.04 | 1.59 | .23 | .18 |
| Kurtosis | .24 | -.05 | 4.46 | -.28 | .18 |
| Minimum | -1.00 | -1.00 | -.28 | -.43 | -1.00 |
| Maximum | 1.00 | 1.00 | 1.00 | 0.94 | 1.00 |
|  |  |  |  |  |  |

^a^iCBT: internet-based cognitive behavioral therapy.

^b^Calculated as (Pre - Post)/Pre and then outliers recoded to a maximum of 1.00 or -1.00. Positive difference score indicates improvement (reduction in symptom level); Zero indicates no change; Negative score indicates got worse over time period of program use.

^c^GAD-7: Generalized Anxiety Disorder 7-item scale.

^d^PHQ-9: Patient Health Questionnaire 9-item scale.

^e^SPIN-17: Social Phobia Inventory 17-item scale.

^f^MOS-6: Medical Outcomes Study Sleep 6-item scale.
