# Supplementary material for: Internet-Based Cognitive-Behavioral Therapy for College Students With Anxiety, Depression, Social Anxiety, or Insomnia: Four Single-Group Longitudinal Studies of Archival Commercial Data and Replication of Employee User Study
Source: JMIR Form Res. 2020 Jul 23;4(7):e17712. doi: 10.2196/17712 (PMC7413280; doi:10.2196/17712)
Supplement: Multimedia Appendix 3 [file formative_v4i7e17712_app3.docx]

|  | Total sample *N*=951 | | Percentage reduction in clinical symptoms from Pre to Post  (improvement) |
| --- | --- | --- | --- |
| Lessons used | *n* students | % |  |
| 2 | 426 | 44.8 | 15.7 |
| 3 | 182 | 19.1 | 23.2 |
| 4 | 106 | 11.1 | 24.4 |
| 5 | 57 | 6.0 | 27.0 |
| 6 | 37 | 3.9 | 30.6 |
| 7 | 21 | 2.2 | 26.7 |
| 8 | 122 | 12.8 | 40.4 |
