# Supplementary material for: Internet-Based Cognitive-Behavioral Therapy for College Students With Anxiety, Depression, Social Anxiety, or Insomnia: Four Single-Group Longitudinal Studies of Archival Commercial Data and Replication of Employee User Study
Source: JMIR Form Res. 2020 Jul 23;4(7):e17712. doi: 10.2196/17712 (PMC7413280; doi:10.2196/17712)
Supplement: Multimedia Appendix 4 [file formative_v4i7e17712_app4.docx]

|  | iCBT^a^ Program | | |  |
| --- | --- | --- | --- | --- |
| Factor | Stress, anxiety,  and worry | Depression | Social  anxiety | |
| Count of users | n=323*^b^* | n=335^c^ | n=199^d^ | |
|  |  |  |  | |
| **Lessons used***^e^* | *F_1,322_*=10.14; *P*=.002 | *F_1,334_*=11.68; *P*<.001 | *F_1,198_*<1; *P*=.81 | |
| All 8 Lessons | 45.5 (*n*=38) | 48.3 (*n*=55) | 20.9 (*n*=10) | |
| 3-7 Lessons | 30.6 (*n*=134) | 23.7 (*n*=129) | 15.6 (*n*=107) | |
| 2 Lessons | 23.4 (*n*=151) | 17.5 (*n*=151) | 14.8 (*n*=82) | |
| Ratio All 8 *vs*. 2 | 1.94 | 2.76 | 1.41 | |
|  |  |  |  | |
| **Gender** | *F_1,322_*=8.38; *P*=.004 | *F_1,334_*=3.13; *P*=.08 | *F_1,198_*=4.04; *P*=.05 | |
| Male | 39.7 (*n*=63) | 31.3 (*n*=76) | 20.7 (*n*=70) | |
| Female | 26.6 (*n*=260) | 23.1 (*n*=259) | 12.7 (*n*=129) | |
| Ratio Male *vs.* Female | 1.49 | 1.35 | 1.63 | |
|  |  |  |  | |
| **Age***^e^* | *F_1,322_*<1; *P*=.86 | *F_1,334_*=3.35; *P*=.07 | *F_1,198_*=2.51; *P*=.12 | |
| Older Age (22-62) | 29.5 (*n*=153) | 29.4 (*n*=133) | 18.2 (*n*=84) | |
| College Age (15-21) | 28.9 (*n*=170) | 22.0 (*n*=202) | 13.6 (*n*=115) | |
| Ratio Old *vs.* Young | 1.02 | 1.34 | 1.28 | |
|  |  |  |  | |
| **Coach support** | *F_1,322_*=1.91; *P*=.17 | *F_1,334_*<1; *P*=.53 | *F_1,198_*=2.05; *P*=.15 | |
| Yes | 33.7 (*n*=84) | 27.4 (*n*=75) | 22.2 (*n*=31) | |
| No | 27.6 (*n*=239) | 24.2 (*n*=260) | 14.3 (*n*=168) | |
| Ratio Yes *vs.* No | 1.22 | 1.13 | 1.55 | |
|  |  |  |  | |
| **Teammate support** | *F_1,322_*<1; *P*=.89 | *F_1,334_*=3.79; *P*=.05 | NA*^f^* | |
| Yes | 29.8 (*n*=42) | 32.3 (*n*=71) | NA (*n*=2) | |
| No | 29.1 (*n*=281) | 23.0 (*n*=264) | NA (*n*=197) | |
| Ratio Yes *vs.* No | 1.02 | 1.40 | NA | |
|  |  |  |  | |
| **Duration days of use** *^e^* | *F_1,322_*<1; *P*=.88 | *F_1,334_*<1; *P*=.71 | *F_1,198_*<1; *P*=.74 | |
| Longer – 13 weeks | 28.7 (*n*=111) | 26.0 (*n*=113) | 16.2 (*n*=65) | |
| Middle – 3 weeks | 33.9 (*n*=108) | 25.9 (*n*=109) | 15.0 (*n*=78) | |
| Shorter – 1 week | 24.8 (*n*=104) | 23.0 (*n*=113) | 15.5 (*n*=56) | |
| Ratio Long *vs*. Short | 1.16 | 1.13 | 1.05 | |
|  |  |  |  | |
| **Comprehensive assess** | *F_1,322_*<1; *P*=.95 | *F_1,334_*=1.50; *P*=.22 | *F_1,198_*<1, *P*=.90 | |
| Yes | 29.1 (*n*=286) | 25.8 (*n*=299) | 15.6 (*n*=161) | |
| No | 29.7 (*n*=37) | 18.3 (*n*=36) | 15.2 (*n*=38) | |
| Ratio Yes *vs.* No | 0.98 | 1.41 | 1.03 | |
|  |  |  |  | |
| **Multiple program user** | NA | *F_1,334_*<1; *P*=.61 | NA | |
| Yes | NA (*n*=9) | 19.5 (*n*=24) | NA (*n*=13) | |
| No | NA (*n*=314) | 25.4 (*n*=311) | NA (*n*=186) | |
| Yes *vs.* No Ratio | NA | 0.76 | NA | |

^a^iCBT: internet-based cognitive behavioral therapy.

*^b^*Sample size slightly lower than 325 total as 2 gender diverse students were removed because of too small count of users for reliable sub-group scores.

*^c^*Sample size slightly lower than 347 full count as 12 gender diverse students were removed because of too small count of users for reliable sub-group scores.

*^d^*Sample size slightly lower than 203 total as 4 gender diverse students were removed because of

*^e^F* tests for factors with continuous data type of variable used full variance of measure.

*^f^*Not applicable to test because of too small count of users for reliable sub-group scores.
