# Supplementary material for: Internet-Based Cognitive-Behavioral Therapy for College Students With Anxiety, Depression, Social Anxiety, or Insomnia: Four Single-Group Longitudinal Studies of Archival Commercial Data and Replication of Employee User Study
Source: JMIR Form Res. 2020 Jul 23;4(7):e17712. doi: 10.2196/17712 (PMC7413280; doi:10.2196/17712)
Supplement: Multimedia Appendix 5 [file formative_v4i7e17712_app5.docx]

|  | iCBT^a^ Program | | | |  |
| --- | --- | --- | --- | --- | --- |
| Study | Stress, anxiety, and worry | Depression | Social  anxiety | Insomnia  (sleep) | Combined^b^ |
| Sample size if clinical status at Pre use of program, *n* (% of total) | | | | | |
| College | 325 (34.2) | 347 (36.5) | 203 (21.3) | 76 (8.0) | 951 |
| Employee^c^ | 259 (36.6) | 211 (29.8) | 110 (15.6) | 127 (18.0) | 707 |
| User age in years - mean (SD) | | | | | |
| College | 23.24 (5.51) | 23.21 (6.78) | 23.00 (6.38) | 25.72 (9.06) | 23.79 |
| Employee^c^ | 36.00 (11.28) | 37.83 (11.79) | 37.20 (11.73) | 43.06 (12.51) | 38.53 |
| User gender - female % (*n*) | | | | | |
| College | 80.0 (360) | 74.6 (259) | 63.5 (129) | 76.3 (58) | 73.60 |
| Employee^c^ | 78.4 (203) | 73.5 (155) | 77.3 (85) | 81.1 (103) | 77.58 |
| Comprehensive assessment - yes % (*n*) | | | | | |
| College | 88.0 (286) | 89.0 (309) | 81.3 (165) | 90.8 (69) | 87.28 |
| Employee^c^ | 86.5 (224) | 89.6 (189) | 91.8 (101) | 78.7 (100) | 86.65 |
| Lessons used - mean (SD) | | | | | |
| College | 3.58 (2.03) | 3.80 (2.23) | 3.37 (1.69) | 3.78 (2.37) | 3.63 |
| Employee^c^ | 3.78 (2.14) | 3.73 (2.13) | 3.98 (2.12) | 4.73 (2.49) | 4.06 |
| Duration of use in days - mean (SD) | | | | | |
| College | 46.54 (46.82) | 44.17 (44.77) | 33.34 (38.30) | 30.07 (33.83) | 38.53 |
| Employee^c^ | 41.99 (43.23) | 38.17 (42.08) | 36.71 (43.09) | 33.90 (34.82) | 37.69 |
| Coach live support - yes % (*n*) | | | | | |
| College | 25.8 (84) | 22.8 (79) | 16.3 (33) | 17.1 (13) | 20.50 |
| Employee^c^ | 36.3 (94) | 37.9 (80) | 26.4 (29) | 26.0 (33) | 31.65 |
| Teammate live support - yes % (*n*) | | | | | |
| College | 12.9 (42) | 22.2 (77) | 1.0 (2) | 3.9 (3) | 10.00 |
| Employee^c^ | 14.7 (38) | 17.5 (37) | 6.4 (7) | 1.6 (2) | 10.05 |

^a^iCBT: internet-based cognitive behavioral therapy.

^b^Average of four programs unweighted by sample size differences between programs.

^c^From [26]; data re-analyzed from clinical status group of employee users in each program.
