# Supplementary material for: Internet-Based Cognitive-Behavioral Therapy for College Students With Anxiety, Depression, Social Anxiety, or Insomnia: Four Single-Group Longitudinal Studies of Archival Commercial Data and Replication of Employee User Study
Source: JMIR Form Res. 2020 Jul 23;4(7):e17712. doi: 10.2196/17712 (PMC7413280; doi:10.2196/17712)
Supplement: Multimedia Appendix 6 [file formative_v4i7e17712_app6.docx]

|  | iCBT^a^ program | | | |  |
| --- | --- | --- | --- | --- | --- |
| Study | Stress, anxiety, and worry | Depression | Social  anxiety | Insomnia  (sleep) | Combined  Total or Average^b^ |
| Sample size if clinical status at pre use of program (*n*) | | | | | |
| College | 325 | 347 | 203 | 76 | 951 |
| Employee^c^ | 259 | 211 | 110 | 127 | 707 |
| Changed to sub-clinical level at post - % (*n*) | | | | | |
| College | 45.8 (149) | 35.7 (124) | 22.2 (45) | 43.4 (33) | 36.8 |
| Employee^c^ | 47.5 (123) | 48.3 (102) | 35.5 (39) | 55.9 (71) | 46.8 |
|  | GAD-7^d^ | PHQ-9^e^ | SPIN-17^f^ | MOS-Sleep-6^g^ |  |
| Average clinical symptom score at Pre and Post for employees^ch^ | | | | | |
| Pre - M (SD)  Post - M (SD) | 14.57 (3.38)  9.49 (5.34) | 15.80 (4.26)  10.39 (6.10) | 40.90 (8.59)  33.15 (13.41) | 59.46 (9.82)  43.06 (13.74) |  |
| Reduction in average clinical symptom score at Post | | | | | |
| College | 29.7 | 24.9 | 15.6 | 22.2 | 23.1 |
| Employee^c^ | 34.9 | 34.2 | 18.9 | 27.6 | 28.9 |

^a^iCBT: internet-based cognitive behavioral therapy.

^b^Average of four programs unweighted by sample size differences between programs.

^c^From [26]; data re-analyzed from clinical status subgroups of employee users in each program.

^d^GAD-7: Generalized Anxiety Disorder 7-item scale.

^e^PHQ-9: Patient Health Questionnaire 9-item scale.

^f^SPIN-17: Social Phobia Inventory 17-item scale.

^g^MOS-Sleep-6: Medical Outcomes Study Sleep 6-item scale.

^h^College sample *M* and SD for Pre and Post by program is in Table 3.
