# Supplementary material for: Internet-Based Cognitive-Behavioral Therapy for College Students With Anxiety, Depression, Social Anxiety, or Insomnia: Four Single-Group Longitudinal Studies of Archival Commercial Data and Replication of Employee User Study
Source: JMIR Form Res. 2020 Jul 23;4(7):e17712. doi: 10.2196/17712 (PMC7413280; doi:10.2196/17712)
Supplement: Multimedia Appendix 7 [file formative_v4i7e17712_app7.docx]

| iCBT^a^ Program  Measure | Severity level | Study Sample | |
| --- | --- | --- | --- |
|  |  | College | Employee^b^ |
| Stress, anxiety, and worry  GAD-7^c^ | Total clinical *N* | 325 | 259 |
|  | Severe - % (*n*) | 47.4 (154) | 51.4 (133) |
|  | Moderate - % (*n*) | 52.6 (171) | 48.6 (127) |
|  |  |  |  |
| Depression  PHQ-9^d^ | Total clinical *N* | 347 | 211 |
|  | Severe - % (*n*) | 23.0 (80) | 19.0 (40 |
|  | Moderately severe - % (*n*) | 44.7 (155) | 39.3 (83) |
|  | Moderate - % (*n*) | 32.3 (112) | 41.7 (88) |
|  |  |  |  |
| Social anxiety  SPIN-17^e^ | Total clinical *N* | 203 | 110 |
|  | Very severe - % (*n*) | 21.7 (44) | 13.6 (15) |
|  | Severe - % (*n*) | 42.4 (86) | 38.2 (42) |
|  | Moderate - % (*n*) | 36.0 (73) | 48.2 (53) |
|  |  |  |  |
| Insomnia (sleep) MOS-Sleep-6^f^ | Total clinical *N* | 76 | 127 |
|  | Severe - % (*n*) | 44.7 (34) | 51.2 (65) |
|  | Moderate - % (*n*) | 55.3 (42) | 48.8 (62) |

^a^iCBT: internet-based cognitive behavioral therapy.

^b^From [26]; data re-analyzed from clinical status subgroups of employee users in each program.

^c^GAD-7: Generalized Anxiety Disorder 7-item scale.

^d^PHQ-9: Patient Health Questionnaire 9-item scale.

^e^SPIN-17: Social Phobia Inventory 17-item scale.

^f^MOS-Sleep-6: Medical Outcomes Study Sleep 6-item scale.
